# Temperature, Humidity, and Latitude Analysis to Estimate Potential Spread and Seasonality of Coronavirus Disease 2019 (COVID-19)

**DOI:** 10.1001/jamanetworkopen.2020.11834

**Published:** 2020-06-11

**Authors:** Mohammad M. Sajadi, Parham Habibzadeh, Augustin Vintzileos, Shervin Shokouhi, Fernando Miralles-Wilhelm, Anthony Amoroso

**Affiliations:** 1 Institute of Human Virology, University of Maryland School of Medicine, Baltimore; 2 Global Virus Network, Baltimore, Maryland; 3 Persian BayanGene Research and Training Center, Shiraz University of Medical Sciences, Shiraz, Iran; 4 Earth System Science Interdisciplinary Center, University of Maryland, College Park; 5 Infectious Diseases and Tropical Medicine Research, Shaheed Beheshti University of Medical Sciences, Tehran, Iran; 6 Department of Atmospheric and Oceanic Science, University of Maryland, College Park; 7 The Nature Conservancy, Arlington, Virginia

## Abstract

**Importance:**

Coronavirus disease 2019 (COVID-19) infection has resulted in a global crisis. Investigating the potential association of climate and seasonality with the spread of this infection could aid in preventive and surveillance strategies.

**Objective:**

To examine the association of climate with the spread of COVID-19 infection.

**Design, Setting, and Participants:**

This cohort study examined climate data from 50 cities worldwide with and without substantial community spread of COVID-19. Eight cities with substantial spread of COVID-19 (Wuhan, China; Tokyo, Japan; Daegu, South Korea; Qom, Iran; Milan, Italy; Paris, France; Seattle, US; and Madrid, Spain) were compared with 42 cities that have not been affected or did not have substantial community spread. Data were collected from January to March 10, 2020.

**Main Outcomes and Measures:**

Substantial community transmission was defined as at least 10 reported deaths in a country as of March 10, 2020. Climate data (latitude, mean 2-m temperature, mean specific humidity, and mean relative humidity) were obtained from ERA-5 reanalysis.

**Results:**

The 8 cities with substantial community spread as of March 10, 2020, were located on a narrow band, roughly on the 30° N to 50° N corridor. They had consistently similar weather patterns, consisting of mean temperatures of between 5 and 11 °C, combined with low specific humidity (3-6 g/kg) and low absolute humidity (4-7 g/m^3^). There was a lack of substantial community establishment in expected locations based on proximity. For example, while Wuhan, China (30.8° N) had 3136 deaths and 80 757 cases, Moscow, Russia (56.0° N), had 0 deaths and 10 cases and Hanoi, Vietnam (21.2° N), had 0 deaths and 31 cases.

**Conclusions and Relevance:**

In this study, the distribution of substantial community outbreaks of COVID-19 along restricted latitude, temperature, and humidity measurements was consistent with the behavior of a seasonal respiratory virus. Using weather modeling, it may be possible to estimate the regions most likely to be at a higher risk of substantial community spread of COVID-19 in the upcoming weeks, allowing for concentration of public health efforts on surveillance and containment.

## Key Points

**Question:**

Is severe acute respiratory syndrome coronavirus 2 associated with seasonality, and can its spread be estimated?

**Findings:**

In this cohort study of 50 cities with and without coronavirus disease 2019 (COVID-19), areas with substantial community transmission of COVID-19 had distribution roughly along the 30° N to 50° N latitude corridor with consistently similar weather patterns, consisting of mean temperatures of 5 to 11 °C combined with low specific and absolute humidity.

**Meaning:**

In this study, the distribution of substantial community outbreaks of COVID-19 along restricted latitude, temperature, and humidity measurements were consistent with the behavior of a seasonal respiratory virus; with modeling, it may be possible to estimate areas at high risk of substantial community transmission of COVID-19.

## Teaser

This cohort study examines the association between climate and the spread of coronavirus disease 2019 (COVID-19) infection.

## Introduction

A substantial number of infectious diseases display seasonal patterns in their incidence, including human coronaviruses. Betacoronaviruses, such as Middle East respiratory syndrome coronavirus (MERS-CoV) and severe acute respiratory syndrome coronavirus (SARS-CoV), are not thought to be seasonal. A burden for health care systems around the globe, influenza is the characteristic example of a seasonal disease.^1^ The incidence of influenza shows substantial seasonal fluctuation in temperate regions of the world but nevertheless displays less seasonality in tropical areas.^2,3,4^ Despite the multitude of possible mechanisms proposed to explain this variation, our current understanding of this phenomenon is still incomplete.^5^

Coronavirus disease 2019 (COVID-19), caused by SARS-CoV-2, initially came to attention in a series of patients with pneumonia of unknown etiology in Wuhan, Hubei province, China, and subsequently spread to many other regions in the world through global travel.^6^ Because of geographic proximity and substantial travel connections, epidemiological modeling of the epicenter estimated that regions in Southeast Asia, specifically Bangkok, would follow Wuhan in the epidemic.^7,8^ However, in reality, the number of cases in the subsequent days in these regions remained low as the epicenter shifted to other countries in Asia, Europe, and North America. More recently, the World Health Organization has declared COVID-19 a pandemic. For many, the biggest concern is not only the swift spread of the pandemic but also how it will behave in the coming months and which areas and populations are most at risk.

A number of studies, including laboratory studies,^9,10^ epidemiological studies,^11,12^ and mathematical modeling,^13^ point to the role of ambient temperature and humidity in the survival and transmission of seasonal respiratory viruses. The tremendous level of research supporting both ambient temperature and humidity in its role in transmission and infection motivated this study to examine the influence of environmental factors on COVID-19. We sought to determine whether climate could be a factor in the spread of this disease.

## Methods

### Study Design

This cohort study examined climate data from 8 cities with substantial community spread of COVID-19 (Wuhan, China; Tokyo, Japan; Daegu, South Korea; Qom, Iran; Milan, Italy; Paris, France; Seattle, US; and Madrid, Spain) (eTable 1 in the Supplement). We compared them with areas that have not been affected or have not had substantial community spread (eTable 2 in the Supplement). This study used a publicly available database and was not considered human participants research according to the US Department of Health and Human Services. This report followed the Strengthening the Reporting of Observational Studies in Epidemiology (STROBE) reporting guideline.

### Main Outcomes and Measures

Substantial community transmission was defined as at least 10 reported deaths in a country as of March 10, 2020. For comparison, we studied cities with and without COVID-19 cases, representing all regions of the globe. For each country, at most 1 representative city was chosen. For countries with COVID-19 cases, we selected locations with community death or, if death rates were not available, community cases; for non–COVID-19 countries, we selected capitals or the largest cities. Community death was defined as community transmission of COVID-19 resulting in death. Temperature analysis was undertaken in a period 30 to 20 days before the first community death to capture a range of days when cases were likely transmitted, based on a reported incubation period of approximately 5 days and a reproduction number of approximately 2.^14,15^ For control cities, the date of the first community death was also used; if this was not available, the date of the first death was used. In countries where there were no deaths, the last date of data collection (ie, March 10, 2020) was used. We obtained COVID-19 country-wide data from the Johns Hopkins Center for Systems Science and Enigeering.^8^ We based 2-m temperatures, relative humidity (RH), specific humidity (Q), and absolute humidity (AH) on data from the European Centre for Medium-Range Weather Forecasts ERA-5 reanalysis.^16^ Two-meter temperature refers to temperature at the height of 2 m above the earth’s surface (ie, the temperature near the earth’s surface, where most human activity takes place); RH is the percentage of the maximum amount of water vapor that the atmosphere can hold at a given temperature (saturation); Q is defined as the mass of water vapor in a unit mass of moist air in grams per kilograms; and AH is defined as the total mass of water vapor present in a given volume or mass of air in grams per meters cubed. Climatologic (1979-2020 data) and persistence forecasting (2019 data) were used to analyze latitude and temperature trends globally and for affected areas using ERA-5.

The first step in weather and climate forecasting is to collect observations of the coupled atmosphere, ocean, and land-surface system (eg, from weather stations and satellites) to initialize the models. Although models are improving constantly, they still suffer from numerical errors and errors introduced by the parameterization of unresolved environmental processes, eg, deep convection and turbulence. Data assimilation techniques (ie, analysis) are used to balance between direct observations and model errors to produce initial states more compatible with the model. To resolve the changing model issue and thus allow for analysis to become a source of gridded observational data sets, Kalnay et al^17^ introduced retrospective analysis (ie, reanalysis), which revisits the entire data set of past direct observations using a frozen version of a model, typically the most recent version. Currently, the most advanced reanalysis product is ERA-5.^16^

### Statistical Analysis

ERA-5 reanalysis climate data are provided on a grid with discretization of approximatively 30 km × 30 km, covering the entire Earth. Preliminary daily updates are available within 5 days of real time, although quality-assured monthly updates are published within 3 months of real time.^16^ We calculated 2-m temperature by interpolating between the lowest model level and the Earth’s surface, taking into account atmospheric conditions. ERA-5 reanalysis data for 2019 was obtained from the Climate Reanalyzer (Climate Change Institute, University of Maine).^18^ ERA-5 reanalysis was also carried out for January to February 2020 and displayed using the Copernicus Climate Change Service Information 2020. The analysis of 2-m temperature was performed in a separate analysis, following the upper air 4-dimensional variational data assimilation analysis.^16^

We used the Mann-Whitney test to compare 2-m temperature, Q, and RH values between cities with and without substantial community transmission. Linear regression analysis was used to determine the association between climate data and the number of cases, with logarithm of total number of cases as a dependent variable; and mean 2-m temperature, mean Q, and mean RH as independent variables. Statistical analysis was performed with Prism version 5 (GraphPad). Statistical significance was set at *P* < .05, and all tests were 2-tailed.

## Results

**Figure 1.**
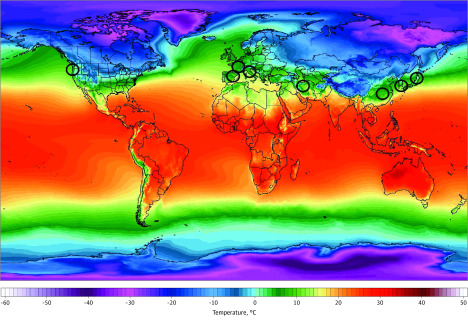
World Temperature Map, November 2018 to March 2019 Color gradient indicates 2-m temperatures. Black circles represent countries with substantial community transmission (ie, ≥10 deaths as of March 10, 2020). Image from Climate Reanalyzer.^18^

Through March 10, 2020, substantial community transmission occurred along a narrow band of latitudes in a consistent east and west pattern. Initially, the new epicenters of disease were all roughly along the 30° N to 50° N, including South Korea (Daegu: 35.9° N; 54 deaths and 7513 cases by March 10), Japan (Tokyo: 35.6° N; 10 deaths and 581 cases by March 10), Iran (Qom: 34.6° N; 291 deaths and 8042 cases by March 10), and Northern Italy (Milan: 45.6° N; 631 deaths and 10 149 cases by March 10) (Figure 1; eTable 1 and eTable 2 in the Supplement).^8^ After the unexpected emergence of a large outbreak in Iran, we made this map in late February. Since then, new areas with substantial community transmission include the northwestern United States (Seattle: 47.5° N; 28 deaths and 959 cases), Spain (Madrid: 40.5° N; 35 deaths and 1695 cases), and France (Paris: 48.7° N; 33 deaths and 1784 cases) (Figure 1; eTable 1 and eTable 2 in the Supplement). During the same period, COVID-19 failed to spread substantially to countries immediately north (eg, Moscow, Russia: 56.0° N; 0 deaths and 10 cases) and south of China, where Wuhan (30.8° N) had 3136 deaths and 80 757 cases. The number of patients and reported deaths in Southeast Asia is much lower compared with more the temperate regions previously mentioned (eg, Bangkok, Thailand: 13.7° N; 1 death and 53 cases; Hanoi, Vietnam: 21.2° N; 0 deaths and 31 cases).^8^

**Figure 2.**
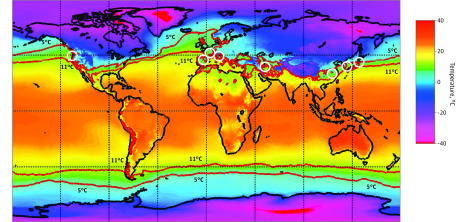
World Temperature Map, January 2020 to February 2020 Color gradient indicates 2-m temperatures, based on data from the European Centre for Medium-Range Weather Forecasts ERA-5 reanalysis. White circles represent countries with substantial community transmission (ie, ≥10 deaths as of March 10, 2020), and red isolines indicate areas with temperatures between 5 and 11 °C. Generated using Copernicus Climate Change Service Information 2020.

**Figure 3.**
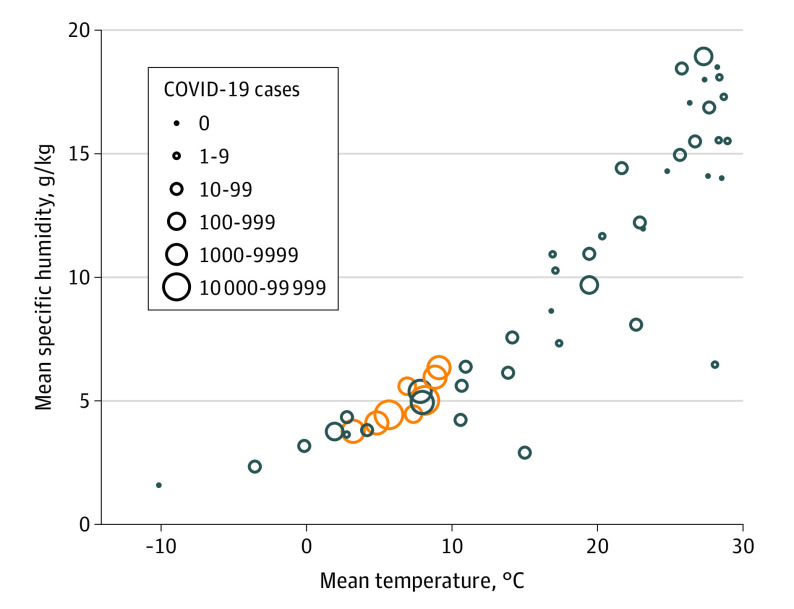
Temperature vs Humidity Plot for 50 Cities With and Without COVID-19 Temperatures and specific humidity are mean values obtained from cities between 20 and 30 days before first community death for cities with substantial community outbreaks of COVID-19. Other cities with and without COVID-19 outbreaks were similarly analyzed, with benchmarks being first community spread–related death (when available) or last day of data collection (March 10, 2020). Orange circles represent countries with substantial community transmission (≥10 deaths as of March 10, 2020), and circle size represents total cases in each country. eTable 2 in the Supplement has characteristics of the 50 cities included.

**Figure 4.**
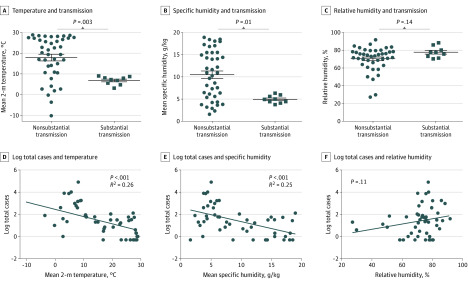
Comparison of Mean Temperature and Humidity Between Cities and Countries With COVID-19 A-C, Mean 2-m temperature, mean specific humidity, and mean relative humidity were compared with the Mann-Whitney test between cities with and without substantial community transmission. Dots indicate values for cities with nonsubstantial transmission, and squares indicate values for cities with substantial transmission. Substantial community transmission was defined as at least 10 reported deaths in a country as of March 10, 2020. D-F, mean 2-m temperature, mean humidity, and mean relative humidity in representative cities were analyzed by linear regression against log of total cases in 50 different countries with and without COVID-19 (eTable 2 in the Supplement). Countries with 0 cases were assigned 0.5 cases. Circles represent values from individual cities.

Further analysis using 2-m temperatures from 2020 yielded similar results (Figure 2). In January 2020 in Wuhan and February 2020 in the other affected cities, there was a similarity in the measures of mean temperature (4-9 °C at the airport weather stations) (eTable 3 in the Supplement). Mean temperatures from a period of 20 to 30 days before the first community death in the area showed similar temperatures (ie, 3-9 °C at the airport weather stations) (eTable 2 and eFigure in the Supplement), and given that city temperatures are slightly higher than airports because of urban effect,^19^ these mean temperatures are within an estimated range of 5 to 11 °C. In addition to having similar mean temperature, these locations also exhibit a commonality in that the timing of the outbreak coincided with a winter nadir in the yearly temperature cycle, with relatively stable temperatures during a period of 1 month or longer, ie, all 8 cities had mean temperatures between 2 and 10 °C for the 3 months between December 2019 and February 2020 (eTable 3 and eFigure in the Supplement). These cities had varying RH (44%-84%) but consistently low Q (3-6 g/kg) and AH (4-7 g/m^3^) (eTable 2 in the Supplement). Having low average temperatures (3-9 °C at airport weather stations) and low Q (4-6 g/kg) tightly clustered the cities with substantial outbreaks as of March 10, 2020, compared with cities that did not have COVID-19 cases (Figure 3). The association between temperature and Q was also statistically significant when comparing cities with and without substantial community spread (*P* = .003 and *P* = .01, respectively) (Figure 4A and B) and when comparing the total cases in their countries with other cities around the world with and without cases (*R*^2^ = .26; *P* < .001 and *R*^2^ = .25; *P* < .001, respectively) (Figure 4D and E). We did not find an association with RH (*P* = .14 and *P* = .11, respectively) (Figure 4C and F).

**Figure 5.**
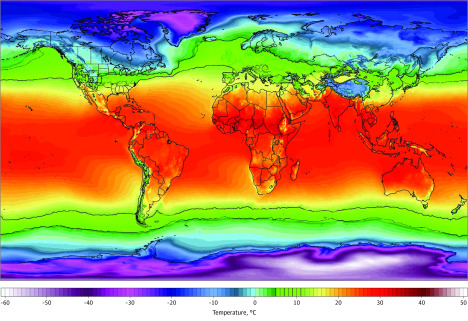
World 2-m Mean Temperature Map, March to April 2019, Estimating At-Risk Zone for March to April 2020 Color gradient indicates mean 2-m temperatures, except neon green band, which shows a zone with temperatures between 5 and 11 °C and specific humidity between 3 and 6 g/kg. The tentative zone at risk for substantial community spread in the near term includes land areas within the neon green bands and will change based on actual mean temperatures during this period and other factors. Image from Climate Reanalyzer.^18^

Given the temporal spread among areas with similar temperature and latitude, some estimations could tentatively be made about the potential community spread of COVID-19 in March and April of 2020. Using 2019 temperature and humidity data for March and April, risk of community spread could be expected to affect areas north of the current areas at risk (Figure 5). These could include (from east to west) Manchuria, Central Asia, the Caucuses, Eastern Europe, Central Europe, the UK, the northeastern and midwestern United States, and British Columbia.

## Discussion

The distribution of the substantial community outbreaks of COVID-19 along restricted latitude, temperature, and humidity measurements were consistent with the behavior of a seasonal respiratory virus. The association between temperature and humidity in the cities affected by COVID-19 deserves special attention. There is a similarity in the measures of mean temperature (ie, 5-11 °C) and RH (ie, 44%-84%) in the affected cities and known laboratory conditions that are conducive to coronavirus survival (4 °C and 20%-80% RH).^20^ In the time we have written up these results, new centers of substantial community outbreaks include parts of Germany and England, all of which had seen mean temperatures between 5 and 11 °C in January and February 2020 and were included in either the January to February 2020 map (Figure 2) or the March to April risk map (Figure 5).

Temperature and humidity are known factors in SARS-CoV, MERS-CoV, and influenza survival.^10,21,22,23,24^ Furthermore, new outbreaks occurred during prolonged periods at these temperatures, perhaps pointing to increased risk of outbreaks with prolonged conditions in this range. Besides potentially prolonging half-life and viability of the virus, other potential mechanisms associated with cold temperature and low humidity include stabilization of the droplet, enhanced propagation in nasal mucosa, and impaired localized innate immunity, as has been demonstrated with other respiratory viruses.^9,10,25,26^ It is important to note that even colder areas in the more northern latitudes have been relatively free of COVID-19, pointing to a potential minimum range for temperature, which could be because of avoidance of freeze-thaw cycles that could affect virus viability or other factors (given that at least 1 human coronavirus tested is freeze-thaw resistant).^27^ Although most studies have focused on RH, this can be affected by temperature, and thus, Q (a measure of absolute humidity) is used to control for this variable. Researchers have found that low Q is a key factor in laboratory transmission of influenza^9^ as well as the onset of seasonal influenza in the US.^12^ All of this points to a potential direct association between temperature and SARS-CoV-2 environmental survival and spreading. This hypothesis can be tested in experimental conditions similar to work that has been done before,^20^ environmental sample testing from areas of ongoing infection, and close epidemiologic and climate studies.

In March to May, temperatures rise dramatically in many areas in the northern hemisphere, which could potentially place many areas at risk according to our simplified model. However, the current model does not consider forecast temperatures or specific humidity, which will be included in future models. The areas to the north that develop temperature profiles overlapping current areas at risk may only do so transiently, as they rapidly warm (with possible exception of areas such as the northwest US and British Columbia, which can stay at yearly nadirs for prolonged periods). Furthermore, as the virus moves further north, it will encounter sequentially less dense human populations. These factors, with climate variables (eg, cloud cover, maximum temperature), human factors (eg, consequences of public health interventions, concentrated outbreaks, such as cruise ships, travel), and viral factors (eg, mutation rate, pathogenesis) not considered or analyzed, suggest that although the current associations with latitude, temperature, and humidity seem strong, a direct causation has not been proven and estimates in the near term have to be considered with extreme caution.

Human coronaviruses (HCoV 229E, HCoV HKU1, HCoV NL63, and HCoV OC43), which usually cause common cold symptoms, have been shown to display strong winter seasonality between December and April and are undetectable in summer months in temperate regions of the northern hemisphere.^28^ Some studies have shown that the alphacoronavirus HCoV 229E peaks in the fall, while HCoV OC43 (a betacoronavirus in the same genera as SARS-CoV-2) has a winter predominance.^29,30^ Although it would be even more difficult to make a long-term estimation at this stage, it is possible that COVID-19 will diminish considerably in affected areas (above 30° N) in the coming months and into the summer. However, given that SARS-CoV-2 is only recently introduced to humans, there is presumably no preexisting immunity. In such cases, whether the 2009 H1N1 influenza pandemic or the first whooping cough pandemics documented in Persia and France in the 1400s and 1500s, the initial epidemic acted unpredictably, so in addition to their recognizable seasonal peak, they had additional peaks outside their later seasonal patterns.^15,31^

The spread of the SARS-CoV-2 virus in the upcoming years could follow different patterns; it could prevail at low levels or cause several seasonal peaks in tropical regions like influenza,^2,3,5^ cause outbreaks in the southern hemisphere at the same time, and begin to rise again in late fall and winter in temperate regions in the upcoming year. Another possibility is that, combined with intensive public health efforts, it will not be able to sustain itself in the summer in the tropics and southern hemisphere and disappear, just as SARS-CoV did in 2003; however, the ever-increasing number of cases worldwide make this increasingly less likely. MERS-CoV has been pointed to as a betacoronavirus that can spread in all seasons. However, it should be remembered that most cases of MERS-CoV were in the Arabian Peninsula and that influenza infection there does not follow the same pattern as in more temperate climates.^32^ In the upcoming summer months in the northern hemisphere, surveillance efforts for SARS-CoV-2 in currently affected areas will be important to determine whether there is a viral reservoir (eg, prolonged stool shedding). Similarly, surveillance efforts in the tropics as well as in New Zealand, Australia, South Africa, Argentina, and Chile between the months of June and September may be of value in determining its establishment in the human population.

An avenue for further research involves the use of integrated or coupled epidemiological-earth-human systems models, which can incorporate climate and weather processes and variables (eg, dynamics of temperature, humidity) and their spatiotemporal changes as well as simulate scenarios of human interactions (eg, travel, transmission due to population density). Such models can assimilate data currently being collected to accelerate the improvements of model estimations on short time scales (ie, daily to seasonally). This approach would allow researchers to explore questions such as which population centers are most at risk and for how long; where to intensify large-scale surveillance and tighten control measures to prevent spreading; how to better understand limiting factors for virus spreading in the southern hemisphere; and how to make estimations for the 2021 to 2022 virus season. A better understanding of the cause of seasonality for coronaviruses and other respiratory viruses would undoubtedly aid in better treatments and/or prevention and be useful in determining which areas need heightened surveillance.

### Limitations

This study has limitations. The reported data for number of cases and mortality are invariably different in different countries, owing to differences in availability of testing, the sensitivity and specificity of each test, and reporting. Other potential factors that influence transmission (eg, other climate factors, public health interventions, travel, population density, air pollution, population demographic characteristics, viral factors) were not included in this study.

## Conclusions

In this study, the distribution of substantial community outbreaks along restricted latitude, temperature, and humidity measurements were consistent with the behavior of a seasonal respiratory virus. Additionally, we have proposed a simplified model that shows a zone that may be at increased risk for COVID-19 spread. Using weather modeling, it may be possible to estimate the regions most likely to be at higher risk of substantial community spread of COVID-19 in the upcoming weeks and months, allowing for a concentration of public health efforts on surveillance and containment.

## Notes

Supplement. **eFigure.** Mean 2-Meter Temperature and Specific Humidity Charts for Cities With Substantial COVID-19 Outbreaks **eTable 1.** City Data Related to Substantial COVID-19 Outbreaks **eTable 2.** Cities and Countries With and Without Substantial COVID-19 Outbreaks as of March 10, 2020 **eTable 3.** November 2019 to February 2020 Monthly Climate Data
