# Supplementary material for: Temperature, Humidity, and Latitude Analysis to Estimate Potential Spread and Seasonality of Coronavirus Disease 2019 (COVID-19)
Source: JAMA Netw Open. 2020 Jun 11;3(6):e2011834. doi: 10.1001/jamanetworkopen.2020.11834 (PMC7290414; doi:10.1001/jamanetworkopen.2020.11834)
Supplement: Supplement. — eFigure. Mean 2-Meter Temperature and Specific Humidity Charts for Cities With Substantial COVID-19 Outbreaks eTable 1. City Data Related to Substantial COVID-19 Outbreaks eTable 2. Cities and Countries With and Without Substantial COVID-19 Outbreaks as of March 10, 2020 eTable 3. November 2019 to February 2020 Monthly Climate Data [file jamanetwopen-3-e2011834-s001.pdf]

Supplementary Online Content

Sajadi MM, Habibzadeh P, Vintzileos A, Shokouhi S, Miralles-Wilhelm F, Amoroso A. Temperature, humidity, and latitude analysis to estimate potential spread and seasonality of coronavirus disease 2019 (COVID-19). *JAMA Netw Open*. 2020;3(6):e2011834. doi:10.1001/jamanetworkopen.2020.11834

eFigure. Mean 2-Meter Temperature and Specific Humidity Charts for Cities With Substantial COVID-19 Outbreaks
eTable 1. City Data Related to Substantial COVID-19 Outbreaks
eTable 2. Cities and Countries With and Without Substantial COVID-19 Outbreaks as of March 10, 2020
eTable 3. November 2019 to February 2020 Monthly Climate Data

This supplementary material has been provided by the authors to give readers additional information about their work.

© 2020 Sajadi MM et al. *JAMA Network Open.*

eFigure. Mean 2-Meter Temperature and Specific Humidity Charts for Cities With Substantial COVID-19 Outbreaks. Traces contain data from November 1, 2019 to the February 29, 2020 obtained from ERA-5 reanalysis. Red line indicates 1st community acquired COVID-19 case that led to death. Two black lines show the window between 20-30 days prior to the first death, presumably when community spread first occurred (assuming a doubling of cases every 5 days, and mortality rate between 2-3%). All cities have stable temperature and specific humidity (roughly a 5-10 degree Celsius or g/kg range) over several months, and values 20-30 days prior are representative of those before and afterwards.

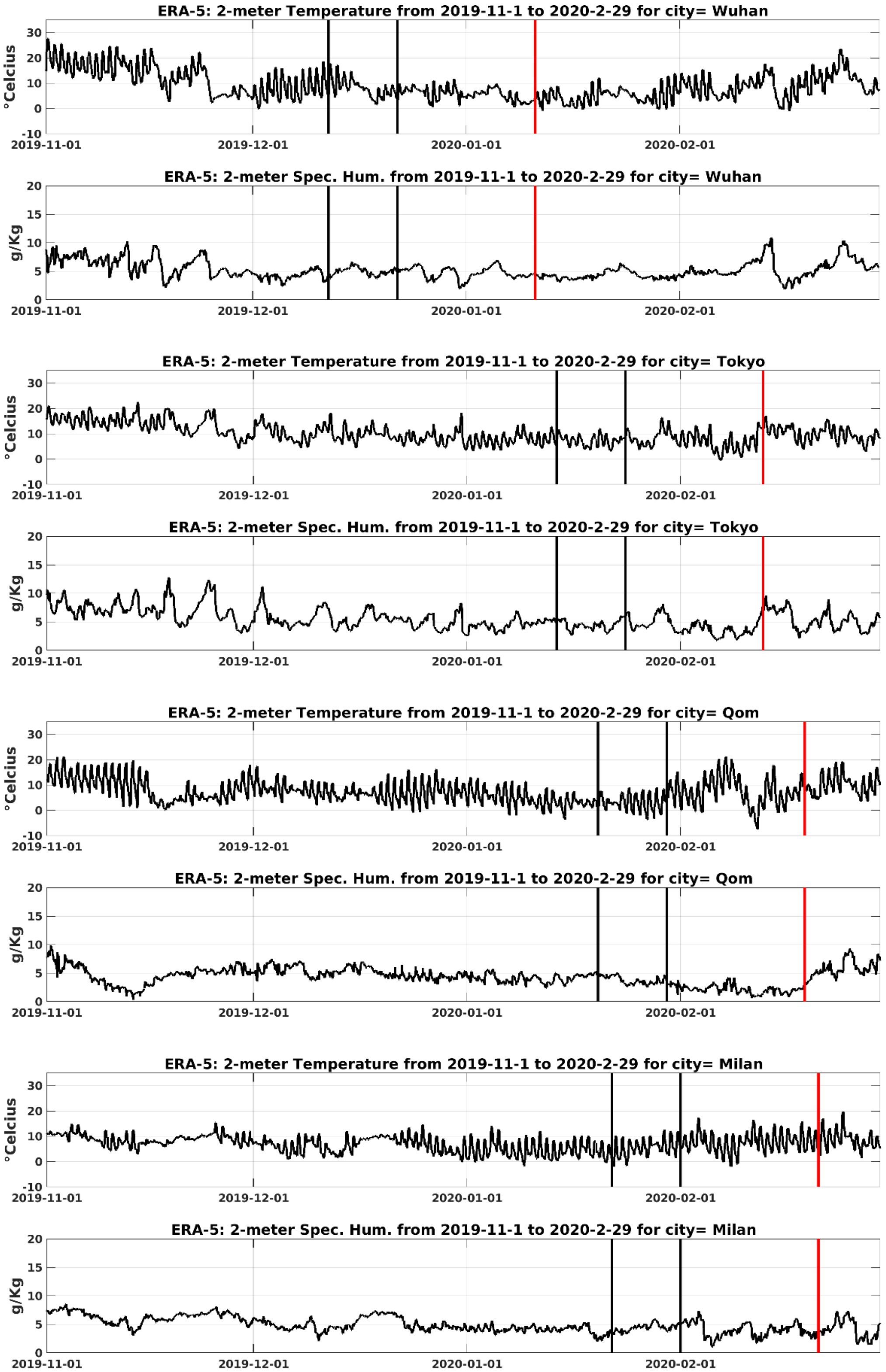

© 2020 Sajadi MM et al. *JAMA Network Open.*

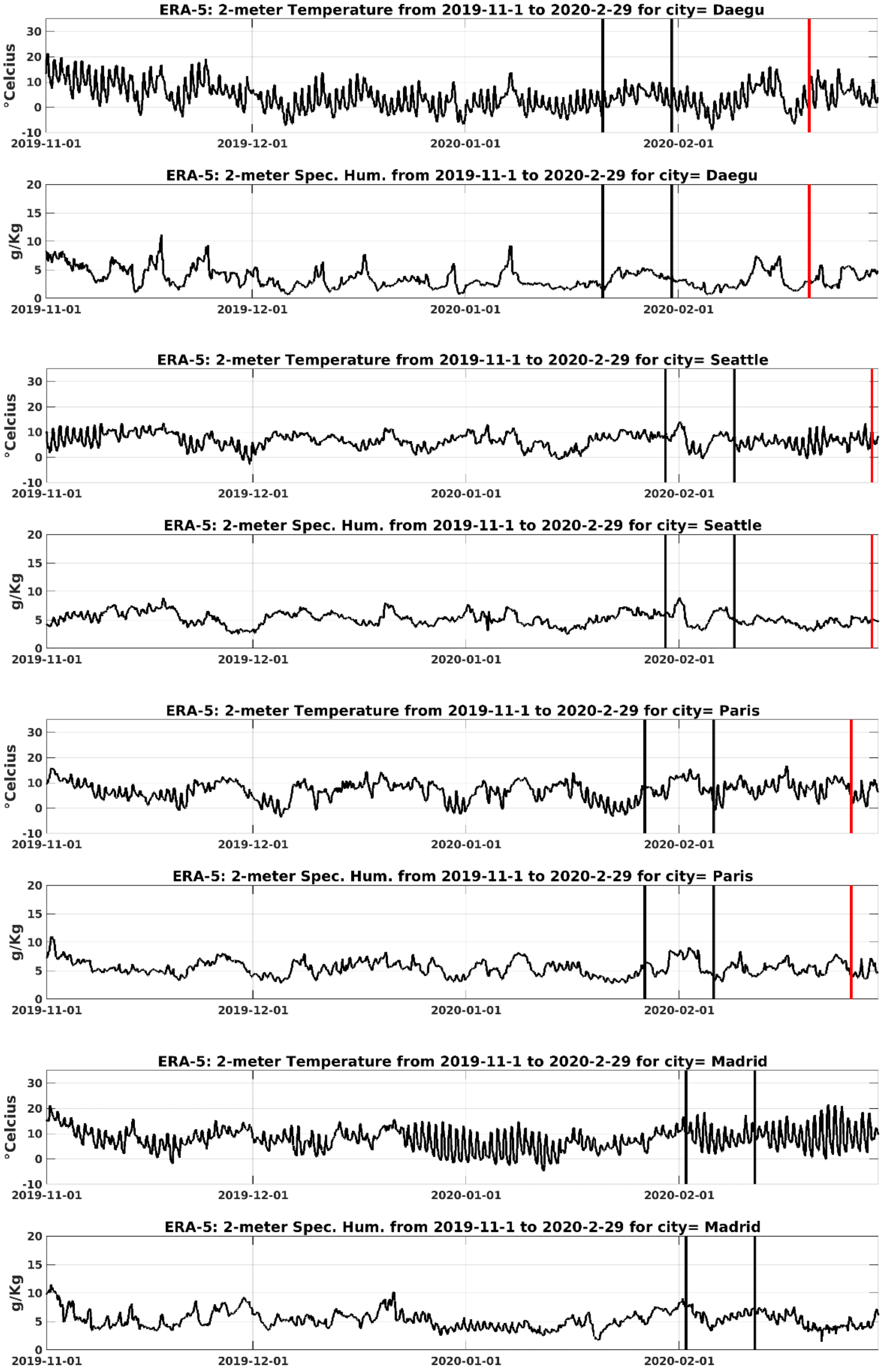

© 2020 Sajadi MM et al. *JAMA Network Open.*

eTable 1. City Data Related to Substantial COVID-19 Outbreaks

**Country (representative city)**	Date of 1st reported community death	Date 1st reported case in country	Latitude (º N')	Koppen Climate Classification
China (Wuhan)	January 11, 2020	December 31, 2019	30·7766	Humid Subtropical Climate (Cfa)
Japan (Tokyo)	February 13, 2020	January 16, 2020	35·5494	Humid Subtropical Climate (Cfa)
S. Korea (Daegu)	February 20, 2020	January 31, 2020	35·8995	Dry-winter humid subtropical climate (Cwa)
Iran (Qom)	February 19, 2020	February 19, 2020	34·5756	Hot desert climate (Bwh)/ Hot semi-arid climates (Bsh)
Italy (Milan)	February 21, 2020	January 31, 2020	45·6301	Humid Subtropical Climate (Cfa)
France (Paris)	February 26, 2020	January 25, 2020	48·7262	Marine west coast climate (Cfb)
USA (Seattle	February 29, 2020	January 20, 2020	47·4502	Mediterranean warm/cool summer climates (Csb)
Spain (Madrid)	March 3, 2020	February 1, 2020	40·4983	Mediterranean hot summer climate (Csa)

© 2020 Sajadi MM et al. *JAMA Network Open.*

eTable 2. Cities and Countries With and Without Substantial COVID-19 Outbreaks as of March 10, 2020

**City**	Country	Time first community death or last day of data collection	Latitude	mean temp	mean specific humid	mean relative humidity	Total country death by 3/10	Total country cases by 3/10
Wuhan	China	1/11/2020	30.7766	8.130788	5.022364	77.30829	3136	80757
Tokyo	Japan	2/13/2020	35.5494	7.353401	4.465458	71.13801	10	581
Qom	Iran	2/19/2020	34.5756	3.201096	3.769332	70.31583	291	8042
Daegu	S. Korea	2/20/2020	35.8995	4.826524	4.110363	75.76329	54	7513
Milan	Italy	2/22/2020	45.6301	5.660805	4.442617	77.54707	631	10149
Paris	France	2/26/2020	48.7262	9.098398	6.348156	85.98263	33	1784
Seattle	USA	2/29/2020	47.4502	6.912893	5.601131	88.38819	28	959
Bangkok	Thailand	3/1/2020	13.69	27.68248	16.87061	74.56445	1	53
Melbourne	Australia	3/1/2020	-37.669	19.44297	9.693879	69.98207	3	107
Madrid	Spain	3/3/2020	40.4983	8.83284	5.964032	80.2157	35	1695
Manilla	Philippines	3/5/2020	14.5123	26.70803	15.50467	71.81731	1	33
London	UK	3/9/2020	51.5048	7.818374	5.393922	79.59356	6	382
Heinsberg	Germany	3/9/2020	51.0505556	7.960705	4.939414	73.19447	2	1457
Oslo	Norway	3/10/2020	60.1976	1.925947	3.764204	79.56566	0	400
Cairo	Egypt	3/10/2020	30.1128	13.86202	6.145834	62.86863	1	59
Solo	Indonesia	3/10/2020	-7.5155	25.80165	18.44953	86.78888	0	27
Toronto	Canada	3/10/2020	43.6777	-3.55639	2.342968	73.53794	1	79
Moscow	Russia	3/10/2020	55.9736	-0.1676	3.183509	82.89326	0	10
Bogota	Columbia	3/10/2020	4.6972	16.90955	10.93645	71.57865	0	3
Sao Paulo	Brazil	3/10/2020	-23.4306	21.6606	14.42052	82.90138	0	31
Helsinki	Finland	3/10/2020	60.321	2.770063	4.349124	91.77417	0	40
Baghdad	Iraq	3/10/2020	33.267	10.58592	4.237373	51.61052	7	71
Mexico City	Mexico	3/10/2020	19.4361	17.36212	7.336186	50.13445	0	7
Havana	Cuba	3/10/2020	23.1136	24.79295	14.30441	75.47668	0	0
Managua	Nicaragua	3/10/2020	12.1447	28.54474	14.01472	58.49455	0	0
San Salvador	El Salvador	3/10/2020	13.4448	27.60709	14.10288	60.82296	0	0
Buenos Aires	Argentina	3/10/2020	-34.82	22.9162	12.22035	70.68398	1	17
Santiago	Chile	3/10/2020	-33.3969	22.66342	8.086184	47.22174	0	13
Asunción	Paraguay	3/10/2020	-25.2415	28.68036	17.30989	70.40358	0	1
Montevideo	Uruguay	3/10/2020	-34.7326	23.13664	11.96479	68.53096	0	0
Riyadh	Saudi Arabia	3/10/2020	24.958202	15.00962	2.908044	27.17325	0	20
Dakar	Senegal	3/10/2020	14.6709	28.07748	6.469012	29.83022	0	4
Lagos	Nigeria	3/10/2020	6.465422	28.38798	18.09742	76.24715	0	2
Luanda	Angola	3/10/2020	-8.8481	28.24247	18.51496	77.95641	0	0
Addis Ababa	Ethipia	3/10/2020	8.9834	16.8253	8.642159	58.40466	0	0
Maputo	Mozambique	3/10/2020	-25.9237	26.33464	17.06566	80.50979	0	0
Johannesburg	South Africa	3/10/2020	-26.1367	20.32142	11.66638	68.76288	0	7
Athens	Greece	3/10/2020	37.9356	10.67141	5.611168	70.87465	0	89
Warsaw	Poland	3/10/2020	52.4493	4.159489	3.823824	75.15617	0	22
Algiers	Algeria	3/10/2020	36.6975	14.14485	7.57031	77.43974	0	20
Kiev	Ukraine	3/10/2020	50.3382	2.757848	3.640373	78.26205	0	1
Jerusalem	Israel	3/10/2020	31.8631	10.92998	6.390851	75.86891	0	58
Nur-sultan	Kazakstan	3/10/2020	51.0281	-10.1602	1.595096	80.08856	0	0
Mumbai	India	3/10/2020	19.0896	25.66363	14.95476	73.13478	0	56
Hanoi	Vietnam	3/10/2020	21.2187	19.42837	10.95041	75.86271	0	31
Phnom Penh	Cambodia	3/10/2020	11.5527	28.32818	15.54713	66.13229	0	2
Kuala Lampur	Malaysia	3/10/2020	2.7456	27.30214	18.94094	83.6464	0	129
Colombo	Sri Lanka	3/10/2020	7.1802	28.94067	15.52587	63.97944	0	1
Wellington	New Zealand	3/10/2020	-41.3276	17.11309	10.27477	84.77074	0	5
Port Moresby	Papua New Guinea	3/10/2020	-9.441	27.36814	18.00307	78.48321	0	0

© 2020 Sajadi MM et al. *JAMA Network Open.*

eTable 3. November 2019 to February 2020 Monthly Climate Data

City	Nov 2019				Dec 2019				Jan 2020				Feb 2020			
	2m (ºC)	Rh (%)	Q (g/kg)	AH (g/m³)	2m (ºC)	Rh (%)	Q (g/kg)	AH (g/m³)	2m (ºC)	Rh (%)	Q (g/kg)	AH (g/m³)	2m (ºC)	Rh (%)	Q (g/kg)	AH (g/m³)
Cities with significant community transmission of COVID-19																
**Wuhan**	14	66	6	8	8	74	5	6	5	84	4	6	10	77	6	7
**Tokyo**	14	72	7	9	10	73	5	7	8	72	5	6	9	66	5	6
**Daegu**	9	68	5	6	2	62	3	4	3	67	3	4	4	62	3	4
**Qom**	9	61	5	5	7	72	5	6	4	69	4	4	8	44	4	4
**Milan**	9	85	6	8	7	80	5	6	5	77	4	5	8	60	4	5
**Seattle**	7	84	5	7	7	88	5	7	7	85	5	7	6	82	5	6
**Paris**	8	89	6	7	6	87	5	7	6	88	5	7	8	81	6	7
**Madrid**	9	74	6	7	8	77	5	6	6	77	5	5	9	71	5	6
Cities tentatively predicted to be at risk for COVID-19 in March and April 2020																
**London**	7	90	6	7	7	89	5	7	7	89	6	7	7	81	5	6
**Manchester**	6	92	5	7	5	91	5	6	6	90	5	7	6	84	5	6
**Berlin**	6	88	5	7	4	84	4	6	4	84	4	5	6	77	5	6
**Prague**	6	86	5	6	3	82	4	5	2	84	4	5	5	72	4	5
**Hamburg**	6	91	5	7	5	87	5	6	5	89	5	6	6	82	5	6
**Vancouver**	8	81	5	7	6	85	5	6	5	82	5	6	5	81	4	6
**New York**	7	65	4	5	3	74	4	5	3	69	4	4	4	69	4	5
**Warsaw**	6	88	5	7	3	86	4	5	2	87	4	5	4	79	4	5
**Glasgow**	5	88	5	6	6	89	5	7	7	86	5	7	5	85	5	6
**Kiev**	5	84	5	6	3	87	4	5	1	86	3	4	2	77	4	4
**St. Louis**	5	71	4	5	3	75	4	5	2	78	4	4	2	72	3	4
**Beijing**	5	53	3	4	-3	54	2	2	-3	58	2	2	1	62	2	3
Previously predicted city where COVID-19 failed to take hold																
**Bangkok**	28	70	16	19	26	70	15	17	28	74	17	20	28	70	16	19

Average 2m temperature (ºC), relative humidity (RH, %), specific humidity (Q, g/kg), and absolute humidity (AH, g/m³) data from cities with community spreading of COVID-19 (as of 3/10/20). Temperature and humidity based on data from the ECMWF ERA-5 reanalysis. Temperatures not adjusted for urban effect.

© 2020 Sajadi MM et al. *JAMA Network Open.*
